# Hyperthyroidism in Childhood: Causes, When and How to Treat

**DOI:** 10.4274/Jcrpe.854

**Published:** 2013-03-01

**Authors:** Juliane Léger, Jean Claude Carel

**Affiliations:** 1 Université Paris Diderot, Sorbonne Paris Cité; Hopital Robert Debré, Service d'Endocrinologie Diabétologie Pédiatrique et Centre de Référence des Maladies Endocriniennes Rares de la Croissance; Institut National de la Santé et de la Recherche Médicale UMR 676, Paris France

**Keywords:** Hyperthyroidism, childhood, Graves’ disease

## Abstract

Graves’ disease (GD) is the most common cause of hyperthyroidism in children. This review gives an overview and update of management of GD. Antithyroid drugs (ATD) are recommended as the initial treatment, but the major problem is the high relapse rate (30%) as remission is achieved after a first course of ATD. More prolonged medical treatment may increase the remission rate up to 50%. Alternative treatments, such as radioactive iodine or thyroidectomy, are considered in cases of relapse, lack of compliance, or ATD toxicity. Therefore, clinicians have sought prognostic indicators of remission. Relapse risk decreases with longer duration of the first course of ATD treatment, highlighting the positive impact of a long period of primary ATD treatment on outcome. The identification of other predictive factors such as severe biochemical hyperthyroidism at diagnosis, young age, and absence of other autoimmune conditions has made it possible to stratify patients according to the risk of relapse after ATD treatment, leading to improvement in patient management by facilitating the identification of patients requiring long-term ATD or early alternative therapy. Neonatal autoimmune hyperthyroidism is generally transient, occurring in only about 2% of the offspring of mothers with GD. Cardiac insufficiency, intrauterine growth retardation, craniostenosis, microcephaly and psychomotor disabilities are the major risks in these infants and highlight the importance of thyroid hormone receptor antibody determination throughout pregnancy in women with GD, as well as highlighting the need for early diagnosis and treatment of hyperthyroidism.

**Conflict of interest:**None declared.

## INTRODUCTION

**Aetiology of Hyperthyroidism in Childhood**

Hyperthyroidism is a rare but serious disorder in childhood (1), occurring most frequently as a consequence of Graves’ disease (GD), an autoimmune disorder resulting from thyrotropin (TSH) receptor stimulation by autoantibodies. Acute or subacute thyroiditis, chronic lymphocytic thyroiditis, acute or chronic administration of thyroid hormones and/or iodides may also result in transient thyrotoxicosis. McCune-Albright syndrome as well as germline and somatic gain-of-function mutations of the TSH receptor gene, which may be associated with the presence of diffuse hyperplasia and toxic nodules, are also rare causes of thyrotoxicosis, as are TSH-secreting pituitary tumours and thyroid hormone resistance (Table 1).This review will focus on management of GD in childhood and of hyperthyroidism during the fetal and neonatal period.

**Graves’ disease **

**Inc idence**

**Figure 1:**
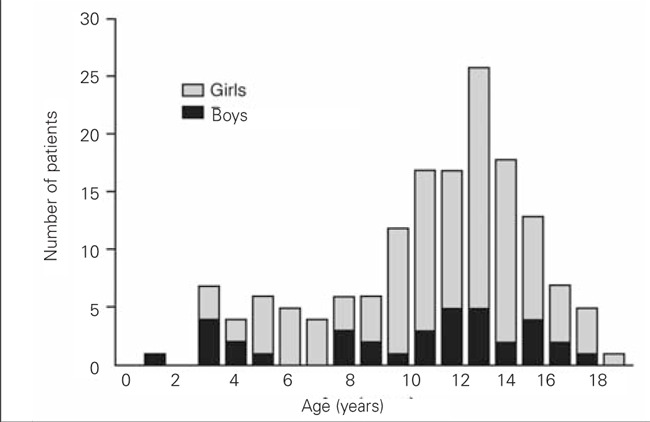
Distribution of patients with Graves’ disease

GD is a rare disease in children, accounting for 1 to 5% of all patients with GD. In adults, this disease affects approximately 2% of women and 0.2% of men (2,3). In both adults and children, GD is much more frequent in female than in male subjects. It may occur at any age during childhood, but it increases in frequency with age, peaking during adolescence (Figure 1). The incidence is thought to be rising and is about 0.1 per 100 000 person-years in young children to 3 per 100 000 person-years in adolescents (1). A frequency of up to 14 per 100 000 patient-years has been reported in Hong-Kong, with no relationship to differences in iodine nutritional status (4,5). GD is more frequent in children with other autoimmune conditions and in children with a familial history of autoimmune thyroid disease.

**Pathogenesis**

The cause of GD remains unclear, but it is believed to result from a complex interaction between genetic background (heredity), environmental factors and the immune system. For unknown reasons, the immune system produces an antibody [TSH receptor antibody (TRAb)] that stimulates the thyroid gland to produce excess thyroid hormone. Genetic susceptibility to the disease is thought to be polygenic. GD has been reported to be associated with the human leukocyte antigen (HLA) gene on chromosome 6p, the cytotoxic T lymphocyte antigen-4 (CTLA-4) gene on chromosome 2q33, and the lymphoid tyrosine phosphatase (PTPN22) gene on chromosome 1p13. Data from twin studies and the higher prevalence of GD in first-degree relatives of patients with this disease than in controls suggest that about 80% of the susceptibility to GD is determined by genetic factors (6,7).

The thyroid-stimulating immunoglobulin (TSI) binds to and stimulates the TSH receptor on the thyroid cell membrane resulting in follicular cell growth, vascularity increase, and in excessive synthesis and secretion of thyroid hormone. The thyroid gland typically displays lymphocytic infiltration, with T-lymphocyte abnormality and absence of follicular destruction. T cells activate local inflammation and tissue remodelling by producing and releasing cytokines, leading to B-cell dysregulation and increase in autoantibody production. An imbalance between pathogenic and regulatory T cells is thought to be involved in both the development of GD and its severity (8).

**Management**

The optimal treatment of GD in childhood remains a matter of debate (9,10,11,12,13), and prospective randomised long-term clinical studies are required to compare treatment failure frequencies and the short- and long-term side effects of the different therapeutic options. Current treatment options include antithyroid drugs (ATD), subtotal or near total thyroidectomy and radioactive iodine (RAI) - I131. There is no specific cure for the disease and each therapeutic option has associated complications. Most patients are initially treated with ATD. However, it is difficult to achieve long-term compliance and the rate of relapse is high. Surgical removal of the thyroid gland and destruction of the gland by RAI treatment are therefore often used as alternatives. Indications for radical treatment in children include relapse after an appropriate course of drug treatment, a lack of compliance on the part of the patient or the parents, and ATD toxicity. As in many rare diseases, there is currently no evidence-based strategy for the management of this disease in children, in contrast to the situation in adults, in whom the disease is more frequent (14,15). GD treatment policy varies considerably within and between countries and depends on local traditions and resources, the age and preference of the patient, the size of the goiter, and the severity of the disease.

Additional treatment with ß blockers (except in patients with asthma or cardiac failure) during the first two weeks of management may help to reduce the patient’s symptoms. This treatment can be given orally twice daily, at a dose of 2 mg/kg/day, and stopped when the patient becomes euthyroid.

**ATD Therapy**

ATD therapy is usually recommended as the initial treatment for hyperthyroidism in children and adolescents. The most commonly used ATDs are carbimazole and its active metabolite, methimazole (MMI) and propylthiouracil (PTU). These drugs inhibit thyroid hormone synthesis by interfering with the thyroid peroxidase-mediated iodination of tyrosine residues in thyroglobulin. PTU can also block the conversion of thyroxine (T4) to triiodothyronine (T3), whereas MMI can not. Both MMI and PTU are associated with minor reactions (rash, urticaria, arthralgia, gastrointestinal problems) in about 5% to 25% of cases. The frequency of agranulocytosis is between 0.2 and 0.5% for both drugs, and other rare but serious side effects include drug-induced hepatitis and production of cytoplasmic anti-neutrophil antibodies. Antibody-positive vasculitis occurs only in exceptional cases. Recently, it has been recommended to avoid the use of PTU because of the high risk of PTU-induced hepatitis and to use only MMI (or carbimazole) as an ATD (16,17). The frequency of side effects may be dose-related and is very small for severe side effects in patients receiving MMI in a dose of less than 10 mg/day (15). Adverse events to MMI were reported to occur within six months of therapy in 90% of patients (18). MMI is also more effective in the short term than PTU (19) and presents a major advantage over PTU in terms of compliance as MMI has a longer half-life and is effective when given as a single daily dose. The initial starting dose of MMI (or carbimazole) is 0.5 to 1 mg/kg/day, with a maximum dose of 30 mg per day. After two to four weeks, when thyroid hormone secretion is effectively blocked and thyroid hormone levels have normalised, the initial dose is gradually reduced by 30 to 50% (15). No additional benefit accrues from the maintenance of a high dose of ATD administered with replacement doses of levo-T4 (L-T4). Recent studies have even suggested that high-dose therapy may be harmful, because the frequency of side effects is dose-dependent. There is also currently no rationale for the use of L-T4 in combination with ATDs to enhance remission rates (15). GD remission on ATD therapy is linked to the restoration of euthyroidism rather than the immunosuppressive effects of the drugs. Hyperthyroidism itself has been shown to worsen the autoimmune aberration, and autoimmunity leads to the generation of more TRAb and worsening of hyperthyroidism. Once this cycle is broken by ATD treatment rendering the patient euthyroid or by surgery, the patient may experience gradual remission of the disease (20). More prolonged use of ATD (at least 2-4 years) in children than in adults may be required to achieve remission. Compliance is therefore an important issue in the management of these children and should be improved by educational strategies. However, the inhibition of autoantibodies obtained on treatment is difficult to predict, probably because the treatment does not target B cells or autoantibodies directly. B lymphocytes are important self-antigen-presenting cells and precursors of antibody-secreting plasma cells. Temporary B-lymphocyte depletion with the monoclonal antibody rituximab may therefore efficiently decrease or abolish the production of TRAb. Large clinical trials of such treatment are currently required (21,22).

Less than 30% of children achieve lasting remission after about 24 months of ATD (23,24,25,26,27). Near-total thyroidectomy and RAI therapy are the definitive therapeutic options, but both carry a high risk of permanent hypothyroidism. However, hypothyroidism is preferable to hyperthyroidism as it is easier to treat, and hyperthyroidism is associated with serious morbidities such as cardio-vascular diseases and osteopenia. Unfortunately, prospective randomised trials are still lacking to evaluate the efficacy of short- and long-term ATD therapy to increase the remission rate in children, and further studies are required to increase our knowledge of ATD treatment in children.

**Surgical Treatment**

Total (or near-total) thyroidectomy is often currently preferred to subtotal (or partial) thyroidectomy to reduce the risk of recurrent hyperthyroidism (11). The vascularity of the gland is decreased by adding iodine to ATD (5 to 10 drops of lugol solution) for one week before surgery (28). L-T4 replacement therapy should be initiated within days of surgery and the patient should be subject to long-term follow-up. Complications such as hypoparathyroidism, vocal cord palsy due to recurrent laryngeal nerve injury, and keloid formation are relatively rare when the operation is performed by a paediatric surgeon with extensive experience and are estimated at about 15%. For patients with recurrent hyperthyroidism after surgery, RAI treatment is recommended because the risk of complications is higher for a second operation (11).

Among radical options, surgery is less commonly used than RAI as a first-line choice and is often recommended only in patients with a large goiter or with ophthalmopathy. For other cases, however, there is still some debate about whether RAI treatment or surgical ablation should be preferred as the definitive treatment for paediatric GD (10,11).

**Iodine Treatment**

RAI treatment is effective in children with hyperthyroidism due to GD, and most patients can be successfully treated with a single oral dose. Low dose aims to cure hyperthyroidism without resulting in hypothyroidism, but the relapse rate is high. Consequently, larger doses (220 to 275 μCi/g, corresponding to about 250 Gy) should be preferred over smaller doses of I131 (29). Hypothyroidism is likely to occur after treatment, and appropriate doses of L-T4 must therefore be administered throughout the patient's life. If hyperthyroidism persists 3 to 6 months after therapy, retreatment with I131 is indicated. There is no evidence of reproductive dysfunction or higher frequencies of abnormalities in the offspring of treated patients (30). However, RAI is absolutely contraindicated during pregnancy and breastfeeding. RAI should also be avoided in very young children because of an increased potential risk of neoplasia. Concerns about potential thyroid malignancy, hyperparathyroidism and high mortality rates have highlighted the need for a large, randomised control study with long-term follow-up to settle this issue definitively (31). 

**Long-term Outcome**

While ATD treatment results in long-term remission in about 40 to 60% of adult patients, less than 30% of children treated with ATDs for an average of two years achieve remission lasting at least two years (23,24,25,26,27,32). identification of patients requiring long-term ATD or early radical treatment. Previous stConsequently, the overall frequency of relapse after a first course of about 2 years of ATD treatment is higher in children than in adults and may reach frequencies as high as 70 to 80%. About 75% of patients relapse within six months of the end of drug treatment, whereas only 10% relapse after 18 months. Methods for identifying the patients who are unlikely to have remission after drug treatment would greatly improve patient management, as they would facilitate the udies had their limitations but evaluated age, goiter size, decrease in body mass index and severity of biochemical hyperthyroidism at onset, TRAb levels at onset and at the end of treatment, and duration of medical treatment as predictive markers of GD relapse during childhood (23,27,33,34,35,36,37). However, all these studies but one (37) were retrospective and none has led to widespread changes in clinical practice. Our prospective study (38) decshowed that the risk of relapse after a first course of ATD for about 2 years increases with non-Caucasian origin, young age, and the severity of the disease at diagnosis as demonstrated by high serum TRAb and free T4 (fT4) levels. Conversely, relapse risk reased with duration of first course of ATD as every additional year of treatment was associated with a decrease in relapse rate. These results highlight the positive impact of a long period of primary ATD treatment on outcome to minimize thyroid autoimmunity and recurrence of the disease. In our study, a prognostic score was constructed allowing the identification of three different risk groups at diagnosis. This type of score would greatly improve patient counseling and therapeutic decision making. However, little is known about long-term outcome, because there have been few studies of the relationship between ATD treatment duration and remission rate or relapse risk in paediatric patients. The need to prescribe longer courses of treatment than in adult patients is widely accepted. Our recent prospective study investigated the effect of ATD treatment duration after three consecutive courses, each lasting for about 2 years (39). With a median study period of 10.4 years, about half the patients achieved remission after the discontinuation of carbimazole. An increase (by a factor of about 2.2) in the predicted remission rate achieved with ATD treatment was related to less severe forms of hyperthyroidism at diagnosis and with the presence of other associated autoimmune conditions. This study suggests that children with GD displaying good compliance with treatment and without major adverse effects of ATD medication may be offered up to 8-10 years of medical treatment with ATD before definitive treatment is envisaged (39). However, continuous treatment, rather than treatment cycles of 2 years, should be considered in future clinical trials. Long-term therapy should also be optimized by educational strategies to improve compliance with treatment and by strategies in medical care, particularly during the transition from paediatric to adult services. Other factors such as genetic background, gender, iodine intake, and smoking are thought to modulate individual responsiveness in adults (32,40,41,42). Large prospective randomised studies in children are therefore required to resolve these issues.

Negative consequences for the patients’ health-related quality of life during and after treatment, even after 14 to 21 years, particularly considering mental performance and vitality, have been demonstrated in adult patients with GD. These problems do not seem to be accounted for by the patient’s thyroid hormone status during follow-up. However, the mode of treatment, whether drug-based, surgical or based on the use of RAI, has been shown to have little impact on health-related quality of life in the long term (43). These aspects have not been studied in children, but it would seem prudent to monitor subjects with hyperthyroidism in childhood in the longer term for neuropsychological, emotional and/or behavioural functioning.

**Neonatal Hyperthyroidism**

**Pathogenesis**

Autoimmune neonatal hyperthyroidism is commonly caused by the passage across the placenta of maternal stimulating antibodies directed against the TRAbs, antibodies which stimulate the adenylate cyclase in foetal thyrocytes leading to thyroid hormone hypersecretion. Hyperthyroidism in pregnancy has a prevalence of approximately 0.2% and is mostly associated with GD (44). Graves’ thyrotoxicosis generally improves in the second half of pregnancy, due to decreases in serum TRAb concentration, but then worsens after delivery (45). In maternal gestational autoimmune GD, the preservation of a normal fetal thyroid hormone state to ensure normal brain development is a complex issue. High levels of antibody transmission are associated with the occurrence of fetal thyrotoxicosis. Fetal hyperthyroidism may develop when fetal TSH receptors become physiologically responsive to TSH and to TRAbs, during the second half of gestation, at around week 20, mostly in women with high levels of TRAbs. It may also occur in the offspring of mothers treated years before for hyperthyroidism who still have circulating TRAbs. Thus, all pregnant women with GD and euthyroid pregnant women with a history of GD should undergo TRAb determinations at the beginning of pregnancy. If TRAbs are detected, the fetus should be considered at risk of developing thyrotoxicosis and monitored accordingly (44,46).

Non-autoimmune neonatal hyperthyroidism due to McCune-Albright syndrome (activating mutation of the Gsα gene) (47) or an activating mutation of the TSH receptor gene is a rare disease. Molecular abnormalities of the TSH receptor, leading to its constitutive activation, may be responsible for severe permanent congenital fetal and postnatal hyperthyroidism. Germline mutations are found in cases of hereditary autosomal dominant hyperthyroidism, and de novo mutations may cause sporadic congenital hyperthyroidism. The clinical course of these diseases requires careful management. Even with high doses of ATDs to control severe congenital thyrotoxicosis, thyroid nodules and goiter enlargement develop early in life, requiring subtotal thyroidectomy followed by RAI therapy (48,49,50).

Transient hyperthyroidism may be observed in pregnant women with a hydatidiform mole. Surgical removal of the mole cures the hyperthyroidism. Familial gestational hyperthyroidism caused by a mutant thyrotropin receptor hypersensitive to human chronic gonadotropin has also been reported in exceptional cases (51). 

**Clinical Manifestations**

Fetal hyperthyroidism precedes neonatal hyperthyroidism. Neonatal autoimmune hyperthyroidism is generally transient, occurring in only about 2% of the offspring of mothers with GD. However, it is associated with a mortality rate of up to 25%, and immediate and long-term morbidity. Fetal and neonatal thyroid function may be disturbed to various extents by the presence of TRAbs, the use of ATDs and the maternal thyroid hormone state. In cases in which maternal disease is untreated or poorly controlled, intrauterine growth retardation, oligoamnios, prematurity, and fetal death commonly occur. Tachycardia, hyperexcitability, poor weight gain contrasting with a normal or large appetite, goiter, stare and/or eyelid retraction and/or exophthalmia, small anterior fontanel, advanced bone age, hepatomegaly and/or splenomegaly are the most frequently observed clinical features during the neonatal period. Cardiac insufficiency is one of the major risks in these infants. Biological abnormalities of the liver may also be observed in the absence of cardiac insufficiency. Craniostenosis, microcephaly, and psychomotor disabilities may occur in severely affected infants (52).

**Diagnosis and Management During Pregnancy and Neonatal Period**

The early diagnosis and treatment of fetal hyperthyroidism or hypothyroidism are crucial and highlight the importance of TRAb determination throughout pregnancy in women with GD. The experience of the ultrasound operator also has an impact on the management of pregnancy in women with GD. Fetal thyroid width and circumference should be evaluated starting from 20 weeks of gestation (53). In fetuses with goiter, the main clinical issue is determining whether the cause is maternal treatment that is appropriate for achieving normal maternal thyroid function but inappropriate and excessive for the fetus leading to fetal hypothyroidism, or whether the problem is associated with fetal thyroid stimulation by maternal GD with the presence of TRAbs causing fetal thyroid stimulation and hyperthyroidism.

Fetal ultrasound scan is a non-invasive tool for detecting fetal thyroid dysfunction. Scans should be taken monthly after 20 weeks of gestation to screen for goiter and/or evidence of fetal thyroid dysfunction in women with GD testing positive for TRAbs and/or receiving ATDs. Thyroid gland enlargement is the starting point for the diagnosis of thyroid dysfunction and ultrasonography is used to assess the presence and vascularity of the goiter. The determination of fetal bone maturation (delayed bone maturation in cases of fetal hypothyroidism) and fetal heart rate (greater than 160/min in cases of foetal hyperthyroidism) may also facilitate the diagnosis of hypo- or hyperthyroidism, guiding the choice of the most appropriate treatment. Invasive fetal blood collection and amniotic fluid sampling are usually not required and should be reserved for cases in which the diagnosis is dubious or intra-amniotic L-T4 injection is required to treat a secondary foetal hypothyroid state (46,54,55,56,57). A combination of maternal criteria (TRAbs titers, ATD use and dose) and fetal criteria (thyroid Doppler signal, fetal heart rate and bone maturation) is used to distinguish between fetal hypothyroidism and hyperthyroidism (57).

The prenatal response to treatment, based on fetal status and on the results of thyroid function tests carried out on cord blood at birth, may validate the prenatal treatment strategy, but probably cannot predict subsequent neonatal thyroid dysfunction (58,59). Remarkably, only a minority of newborns from mothers with gestational autoimmune thyroid disease have a disturbed thyroid hormone state (57,58). Within two to five days of birth, hyperthyroidism may develop in cases in which TRAbs continue to be present in the neonate after the clearance of transplacentally transmitted ATDs from the mother. Thyroid function tests should therefore be repeated in the first week of life, even when normal (or high TSH levels due to excessive ATD in late gestation) results were obtained with cord blood. Strong suspicion of neonatal autoimmune hyperthyroidism, when TRAbs are detectable in cord blood and FT4 levels are high in the two to four days following the birth (FT4 levels >35 pmol/l), should lead to the initiation of ATD treatment in the infant shortly after birth to prevent the development of clinical hyperthyroidism, thereby protecting infants from the serious consequences of this condition (46).

**Treatment**

During gestation, fetal hyperthyroidism can be prevented by administering ATDs to the mother. PTU and MMI both cross the placenta and are equally effective for treating hyperthyroidism in pregnancy (60). However, PTU is the more commonly used of these two drugs as the administration of MMI during organogenesis has been associated with neonatal aplasia cutis (a scalp defect), tracheoesophageal fistula and embryopathy (61). The fetus benefits directly from the maternal ingestion of these drugs, which cross the placenta and act on the fetal thyroid gland. However, these drugs may also expose the fetus to the risk of hypothyroidism, and small doses (usually 100-150 mg PTU or less daily; 10-15 mg MMI or less daily) are therefore recommended. 

During the neonatal period, MMI is preferred (1 mg/kg/day, in three doses). Propranolol (2 mg/kg/day, in two divided doses) can also be needed to control tachycardia during the first one to two weeks of treatment. It is usually possible to decrease the ATD dose progressively, according to thyroid hormone levels. The disease is transient and may last two to four months until TRAbs are eliminated from the infant’s circulation. Mothers can breastfeed while taking ATDs, with no adverse effects on the thyroid status of their infants (62).

## Figures and Tables

**Table 1:**
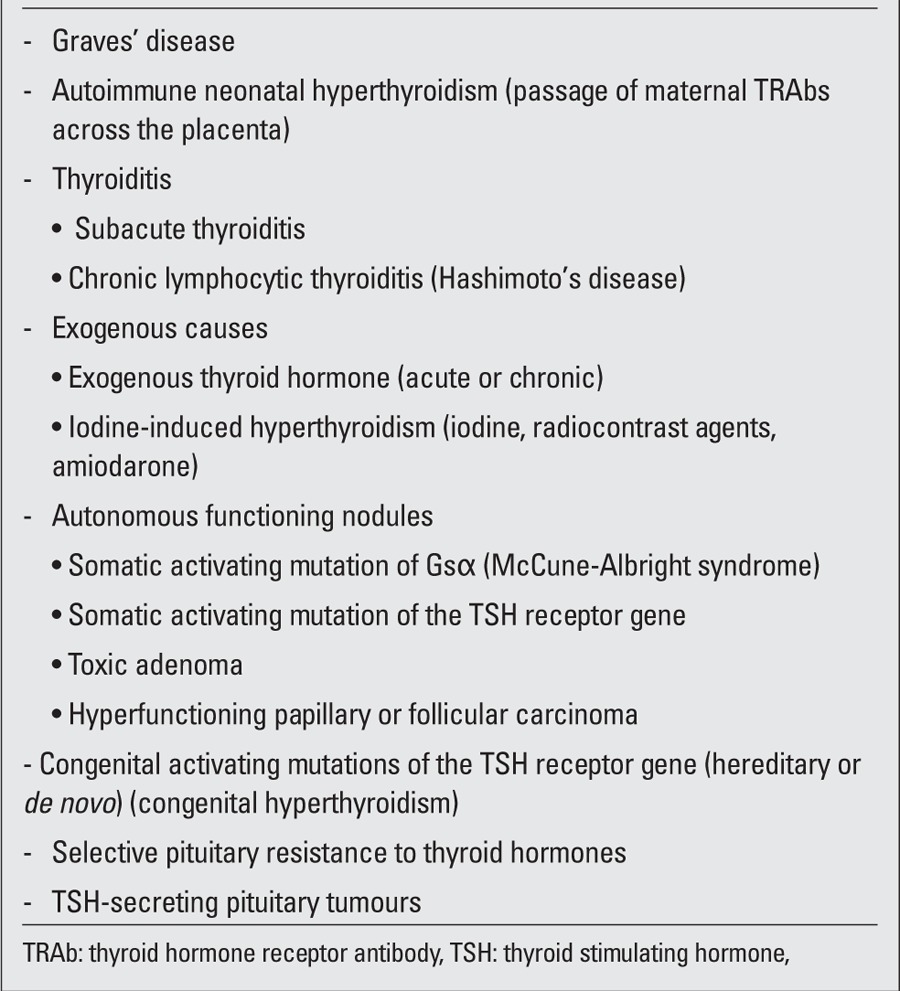
Causes of thyrotoxicosis in children
